# Effects of advance exposure to an animated surgery-related picture book on preoperative anxiety and anesthesia induction in preschool children: a randomized controlled trial

**DOI:** 10.1186/s12887-022-03136-1

**Published:** 2022-02-14

**Authors:** Yanyan Yang, Mazhong Zhang, Ying Sun, Zhezhe Peng, Xiaosu Zheng, Jijian Zheng

**Affiliations:** Department of Anesthesiology, Shanghai Children’s Medical Center, School of Medicine, Shanghai Jiao Tong University, Shanghai, China

**Keywords:** Preoperative anxiety, Picture book, Preschooler

## Abstract

**Background:**

Our aim was to investigate whether early surgical preparation by reading an animated picture book about procedure-related events could reduce the preoperative anxiety in preschoolers.

**Methods:**

131 patients, aged 3–6 years and underwent elective minor surgery were randomized either to a control or a picture book group. Both groups received general information about surgery and anesthesia in pre-anesthesia clinic. Patients in study group also received a surgery-depicting picture book for them to read at home a week earlier before surgery. Child anxiety was evaluated with the modified Yale Preoperative Anxiety Scale Short Form in six observing time points before anesthesia induction, and the compliance of anesthesia induction was assessed with the Induction Compliance Checklist (ICC).

**Results:**

There were significantly lower anxiety scores in picture book group than in control group at the time of ready for intravenous cannulation in operating room [51.9 (23.6) vs. 67.2 (22.0); mean difference 15.3; 95% confidence interval (CI) 6.4–24.1; *P* = 0.001] and at the time of pre-anesthesia visit [27.8 (7.6) vs. 33.2 (13.6); mean difference 5.3; 95%CI 0.93–9.8; *P* = 0.018]. No significant differences of anxiety levels were found between two groups at other observed time points: in the anesthesia outpatient clinic, in the holding area, at separation from parent to operating room (OR), and on entrance to OR (*P* = 0.584, 0.335, 0.228, 0.137, respectively). The percentage of children with poor induction compliance (i.e., ICC ≥ 6) was higher in control group compared with that in picture book group [38% vs.21%; odds ratio(95%CI): 0.78(0.61–0.99); *P* = 0.041].

**Conclusions:**

Home-reading an animated picture book to get familiar with the perioperative events earlier prior to surgery could effectively reduce the preoperative anxiety level and increase the compliance during the induction of anesthesia in preschool children.

**Trial registration:**

ChiCTR2000033583, 06/06/2020 www.chictr.org.cn.

## Background

Approximately 60% of children have experienced significant anxiety before surgery [1]. These children may exhibit distress behaviors such as feeling frightened, crying, grasping to their loved ones tightly, or trying to escape, which may increase the difficulty in anesthesia induction, the risk of emergence agitation, and even long-term behavior abnormalities and psychological disorders [2].

Pharmacological approach is the most commonly used method for mitigating preoperative anxiety. However, medications for sedation are generally administered 30 min before surgery, while children’s anxiety often begins at the time of hospital admission or even much earlier before admission. Also, use of sedatives was associated with potential adverse events [3]. Therefore, many non-pharmacological approaches have been studied and applied clinically to ease the preoperative anxiety. One of those important modalities is preoperative education, which is purposely to improve children's understanding of the condition by providing relevant information, so as to promote children's cooperative participation and reduce preoperative anxiety [4]. Unfortunately, most of those preparation programmes only targeted children over the age of six [5, 6]. By the way, majority of parents of those preschoolers prefer withholding surgical and anesthetic information from their kids for the fears of children’s anxiety and stress induced by potential upcoming surgery and anesthesia. As results, children in this subgroup are psychologically underprepared prior to operating room (OR) [7]. Accordingly, most of these children were likely to develop anxiety when they cognitively perceived that their body would go through some kinds of manipulation under surgical knife when they are forced to put under deep sleep. So provision of developmentally appropriate preparation for preschool children is essential [8]. However, communicating to preschool children about medical treatments is one of very challenging tasks for parents, even for trained doctors.

It was reported that reading an educational comic leaflet before surgery can alleviate preoperative anxiety in children aged six to seventeen years [5], and this practice may not be appropriate for preschoolers since the younger ones’ literacy and cognitive abilities are limited. Picture book, as the main reading material for this age group, plays an important role in their development. Most children enjoy story times with their parents, and in the process of reading they can actively participate and interact with their parents. A study showed that having preschoolers repeatedly visualizing to the vein injection images illustrated in a picture book decreased their behavioral distress scores [9]. Additionally, researchers proposed that as a kind of entertainment education-productions, picture book have an edge over purely educational information like tedious leaflet, because they are associated with amusing stories and children’s best loved cartoon characters [10].

In this study, we hypothesized that the instructive reading of an animated surgery-depicting picture book at home setting before surgery might be a particularly attractive approach in psychological preparation for preschool children to reduce the anxiety. The primary outcome was children’s anxiety measured in the operating room just before intravenous cannulation. The secondary outcomes included children’s compliance during the induction of anesthesia, children’s anxiety during the pre-anesthetic visit and at separation to OR, and parental anxiety.

## Methods

This prospective, randomized and controlled trial was carried out from June, 2020 to March, 2021 after approved by the Institutional Review Board of Shanghai Children's Medical Centre, Shanghai, China on 30 April 2020 (no: SCMCIRB-K2020036-1). This study was registered in the Chinese Clinical Trial Registry (ChiCTR2000033583) on 6 June 2020. Written informed consent was obtained from a parent or legal guardian for each subject. The trial carried out in accordance with the declaration of Helsinki and good clinical practical guidelins, and the authors guaranteed the accuracy and completeness of the data and analysis of this paper.

### Participants

Children, aged 3 to 6 years, ASA physical status I-II, and underwent elective minor surgery under general anesthesia (herniorrhaphy, tonsillectomies etc.) were recruited. The exclusion criteria included parental refusal, significant hearing or visual impairments, developmental delay or neurological diseases, and having any previous surgeries.

### Randomization and blinding

Based on a computer-generated randomization list, participants were assigned either to picture book group or control group in a 1:1 ratio. The group assignment slips were concealed in an envelope, which was opened only by a designated nurse who was not involved other parts of study.

The children and their family were not blinded to the grouping process. The research date collector, anesthesiologists, surgeons and nurses in the operating room were blinded to the group assignment.

### Interventions

The general information about fasting, surgical procedure, risks of anaesthesia, and pain management were given to both groups during their first visit at anaesthesia outpatient clinic within two weeks prior to surgery. In addition, an animated picture book with the illustrated story of perioperative events was mailed to the patient’s home in picture-book group one week before surgery for them to read with their parents.

### The picture book

**Fig. 1:**
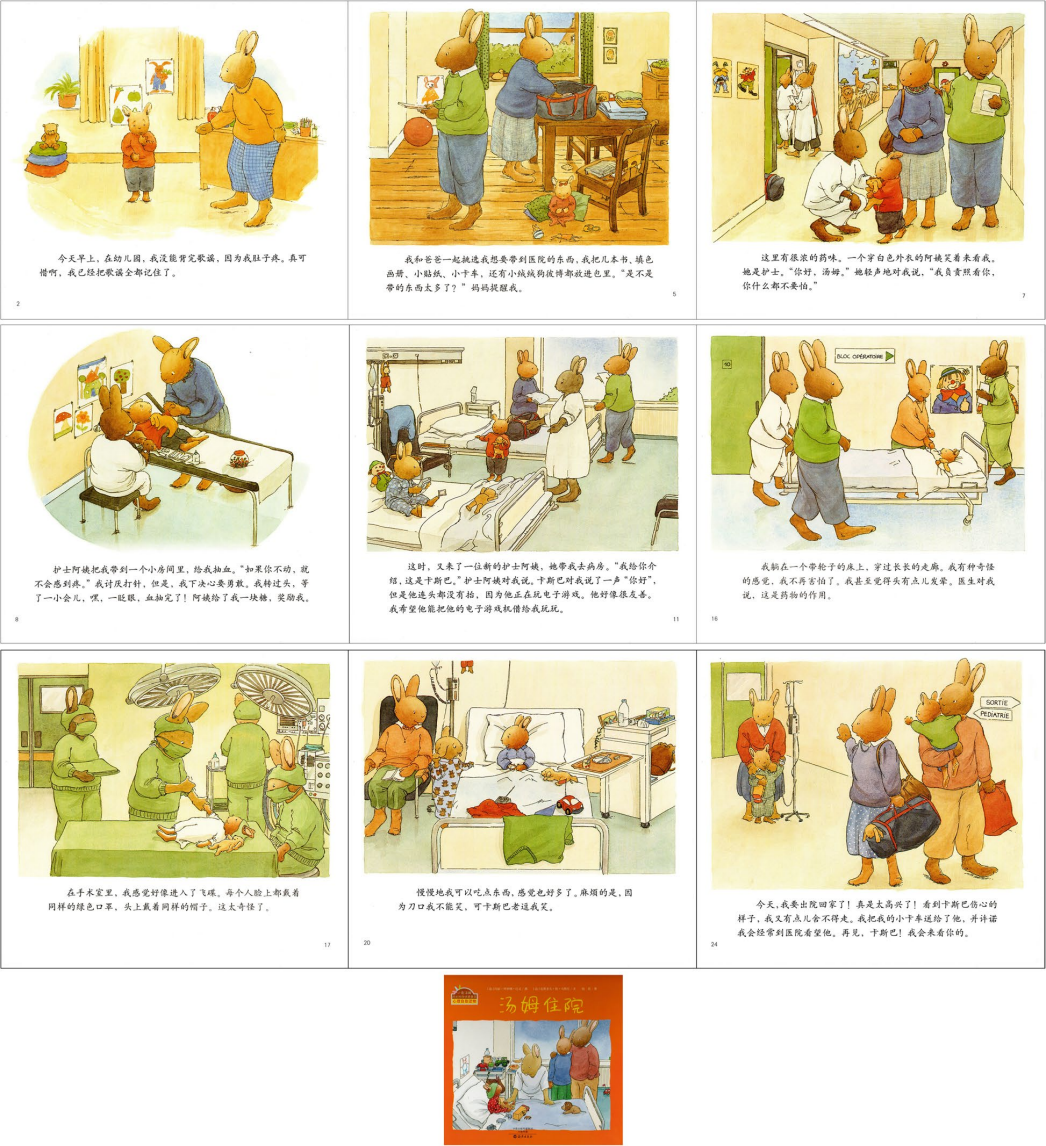
A few pages from “*Tom is in Hospital*” (The picture book is originally in French from a French (https://www.babelio.com/auteur/Christophe-Le-Masne/166238) and Belgium author (https://www.babelio.com/auteur/Marie-Aline-Bawin/96842))

The animated picture book titled “*Tom is in Hospital* “was written by Christophe Le Masne, illustrated by Marie-Aline Bawin and translated by Li Mei. With the help of colorful illustrations and a few of simple and easily understanding words, this book vividly told the perioperative story of a bunny rabbit (named Tom) who had went through. Tom was worried and scared at the beginning for something going to happen to his body, which he never heard of. With his parents’ encouragement and instructive explanation, he took sessions of preoperative psychological preparation and education, started understanding the surgical process step by step, and gradually overcame his fears. With full curiosity and eager to participate, he bravely followed the doctors and walked into the operating room and laid himself on the surgical bed with smile, etc. At last, surgery and anesthesia went through successfully and safely. This book briefly described the events and scenes about surgery and anesthesia in a light and humorous way through a lovely animated character, and gave children the basic information what they expect from the surgery(Fig. 1).

### Study procedures

All participants were assessed for eligibility after the surgery had been confirmed by surgeons in the outpatient clinic. After recruitment, baseline characteristics were collected for both children and parents, such as, birth order, temperament of children (emotionality, activity, sociability, and impulsivity, EASI) [11], and educational level of parents, etc. In addition, general information about anesthesia and surgery was given. Parents in picture book group were also given verbal instruction at outpatient clinic about the book reading protocol.

One week before patients’ hospitalization, the picture books and the detailed reading tips were mailed to study participants’ home. Parents were instructed not to hide the information about the surgery from their children, instead to help kids to get the most basic surgical and anesthetic knowledge through the picture book. Guided by parents, children would go over the series of pictures first to get familiar with the character (Tom) and surgical environment, and so that the children can form a preliminary impression of the operation. Then the child and adult look through those pictures and read the context together at least three times. During this process, parents would ask the child’s thought about this story and provoke the kids for questions, specifically who, what, when, where, why and how, to get the child become more involved in the shared reading session.

The subjects were admitted to the surgical ward in our hospital early on the day of surgery, and were evaluated during a pre-anesthesia visit by an anesthesiologist from the research team. This allowed delivering more details to parents and children about surgery and anesthesia and addressing concerns and answering questions. To make sure the child have basic impression or knowledge about this book, at least three simple questions were asked for kid to answer, such as, “Do you know the name of this lovely ‘rabbit’ (point to the picture)?” “Why did ‘Tom’ come to hospital with his parents?” and “Did ‘Tom’ made more friends in play room with other kids?” “Did you see ‘Tom’ waking-up smiling and happy after surgery” If parents did not follow the reading instruction, or if the child could not answer any one of those questions, the child was seen as not receiving interventions.

### Anesthesia induction and the patients’ compliance

As required, all subjects arrived at the preoperative holding area about 30 min before the anesthesia start time, and no premedication was given. One of parents, in our hospital, was allowed to accompany in the holding area, and parent was separated from his or her child just before OR time started. All patients were induced by intravenous anesthesia. Child’s compliance during the induction of anesthesia was evaluated with the Induction Compliance Checklist (ICC), which was previously developed by Kain and his colleagues and it contains 11 items [11].

### Preoperative anxiety measurements

The modified Yale Preoperative Anxiety Scale Short Form (mYPAS-SF) [12] was used to assess children’s anxiety. The mYPAS-SF is a simplified version of mYPAS, originally developed by Kain et al., and it is more convenient to apply to clinical research settings [13]. This scale contains 18 items in 4 dimensions (activity, emotional expressivity, state of arousal, and vocalization). For each dimension, a blinded investigator recorded the highest scoring behavior witnessed during the observation period [13].

**Fig. 2:**
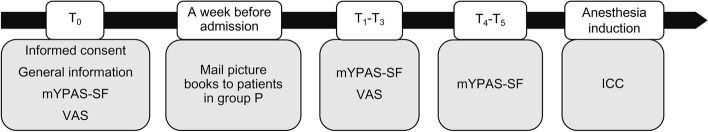
Study timeline T_0_, in the anesthesia outpatient clinic (baseline); T_1_, during the pre-anesthesia visit at surgical ward; T_2_, in the preoperative holding area; T_3_, at separation from parent to OR; T_4_, on entrance to OR; T_5_, the time point of ready for intravenous cannulation; mYPAS-SF, modified Yale Preoperative Anxiety Scale Short Form; VAS, Visual Analogue Scale; ICC, The Induction Compliance Checklist; Group P, picture book group

Children’s anxiety was assessed at the six time points as following: in the anesthesia outpatient clinic (baseline, T_0_), during a pre-anesthesia visit by anesthesiologist shortly after admitted to surgical ward (T_1_), in the preoperative holding area (T_2_), at the time of separation from parent to OR (T_3_), on entrance to OR (T_4_), and at the time of ready for intravenous cannulation (T_5_) (Fig. 2). 

Parental anxiety was measured from T0 to T3 using a visual analogue scale (VAS) (ranging from 1 to 10, “no anxiety” to “extreme anxiety”) [14].

### Outcome variables

The primary outcome was children’s anxiety measured in the OR just before intravenous cannulation. The secondary outcomes included children’s compliance during the induction of anesthesia, children’s anxiety during the pre-anesthetic visit and at separation to OR, and parental anxiety.

### Sample size calculation

Our pilot study reported a mean anxiety score of 63.9 ± 21.9 just before the intravenous anesthesia induction in control group. We expected mYPAS-SF scores in the picture book group to decrease by ≥ 15 points. A sample size calculation of α value of 0.05 and a test power of 90% was performed using the PASS software (Power Analysis and Sample Size software, vision 11.0.7). The minimum of 45 patients in each group was required. To allow for potential post-recruitment drop-out, we initially aimed to recruit 112 participants.

### Statistical analysis

Outcome data were analyzed in the intention to treat (ITT) population. The Shapiro–Wilk test was run to assess the normality. Normally distributed data were reported as mean (standard deviation) and compared with the student’s *t*—test. Non-normally distributed data or ordinal data were presented as median (interquartile range) and compared with Mann–Whitney *U*—test. Categorical data were presented as number (percentage) and compared with the *χ*^*2*^ test or Fisher’s exact test. Changes in anxiety over time were analyzed using generalized estimated equation, with Bonferroni adjustment for multiple tests. All statistical analyses were performed using SPSS 25.0 (SPSS Inc., Chicago, IL). A *P*-value < 0.05 was considered to be statistically significant.

## Results

This prospective, randomized trial was performed in Shanghai Children’s Medical Centre. A total of 131 patients were initially recruited and randomized. Fifteen patients were lost during follow-up and seven children did not follow the reading instruction in picture book group. According to the ITT principle, 116 subjects were analyzed in the end, and 58 in each group. Baseline characteristics were shown in Table 1, and no significant differences were found between the groups.

**Table 1:** Baseline characteristics.

Characteristic	Group P (*n* = 58)	Group C (*n* = 58)
Age, yr, mean(SD)	4.7 (1.0)	4.4 (1.0)
Sex (M/F), *n*	20/38	23/35
Weight, kg, mean(SD)	18.4 (2.5)	18.6 (3.3)
Height, cm, mean(SD)	109.5 (7.4)	106.8 (8.3)
ASA PS (I/II/), *n*	55/3	56/2
Birth order of the child (1/2), *n*	46/12	48/10
EASI, mean(SD)		
Emotionality	14.1 (4.1)	14.7 (3.4)
Activity,	16.6 (3.5)	17.2 (3.7)
Sociability	16.3 (2.6)	16.9 (2.8)
Impulsivity	15.0 (3.1)	14.3 (3.5)
Guardian (F/M/GP), *n*	11/30/17	9/33/16
Highest diploma of guardian, *n* (%)		
Junior high	6(10%)	9(16%)
High school	9(16%)	9(16%)
University	43(74%)	40(69%)

Group P, picture book group; Group C, control group; Sex (M = male; F = female); EASI, emotionality, activity, sociability, and impulsivity; Guardian (F = father; M = mother; GP = grandparents); mYPAS-SF, modified Yale Preoperative Anxiety Scale Short Form

**Fig. 3:**
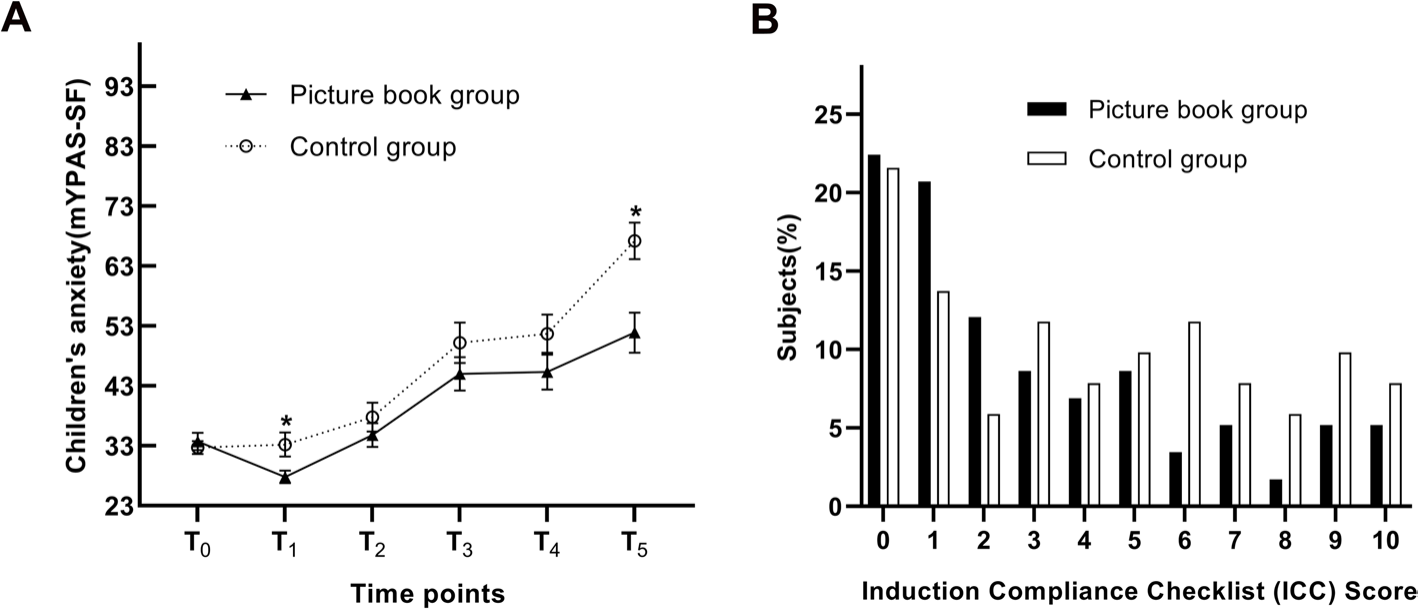
**A**: Changes in anxiety over time by group * *P* < 0.05 between group analyses; mYPAS-SF, modified Yale Preoperative Anxiety Scale Short Form; T_0_, in the anesthesia outpatient clinic (baseline); T_1_, during the pre-anesthesia visit at surgical ward; T_2_, in the preoperative holding area; T_3_, at separation from parent to OR; T_4_, on entrance to OR; T_5_, the time point of ready for intravenous cannulation. **B:** Children’s compliance during induction. ICC, Induction Compliance Checklist

There were significantly lower anxiety scores in picture book group than in control group at the time of ready for intravenous cannulation in operating room (T_5_) [51.9 (23.6) vs. 67.2 (22.0); mean difference 15.3; 95% confidence interval (CI) 6.4–24.1; *P* = 0.001] and at the time of anesthesiologist’s pre-surgery visit after hospital admission(T_1_) [27.8 (7.6) vs. 33.2 (13.6); mean difference 5.3; 95%CI 0.93–9.8; *P* = 0.018]. No significant differences of anxiety levels were found between two groups at other observed time points: in the anesthesia outpatient clinic (T_0_), in the holding area (T_2_), at separation from parent to operating room (T_3_), and on entrance to OR (T_4_) (*P* = 0.584, 0.335, 0.228, 0.137, respectively; Table 2, Fig. 3 A)

**Table 2:** Anxiety level of children and parents

	Group P (n = 58)	Group C (n = 58)	Mean difference (95%CI)	P value
Children’s anxiety (mYPAS-SF)				
T_0_	33.7 (10.4)^bc^	32.7 (5.8)^c^	- 1.0 (-4.5 – 2.6)	0.584
T_1_	27.8 (7.6)^ac^	33.2 (13.6)^c^	5.3 (0.93 – 9.8)	0.018
T_2_	34.8 (14.1)^bc^	37.8 (17.3)^c^	3.0 (-3.1 – 9.1)	0.335
T_3_	44.9 (19.6)^ab^	50.2 (24.3)^ab^	5.2 (-3.3 – 13.8)	0.228
T_4_	45.3 (20.5)^ab^	51.7 (22.8)^ab^	6.4 (-2.0 – 14.8)	0.137
T_5_	51.9 (23.6)^ab^	67.2 (22.0)^abc^	15. 3 (6.4 – 24.1)	0.001
Parental anxiety (VAS)				
T_0_	5(3–5)	4(3–5)	–	0.398
T_1_	4(3–5)	5(4–5)	–	0.921
T_2_	6(5–6)	5(4–6)	–	0.496
T_3_	6(5–7)	6(5–7)	–	0.692

Group P, picture book group; Group C, control group; CI: confidence interval; mYPAS-SF, modified Yale Preoperative Anxiety Scale Short Form; T_0_, in the anesthesia outpatient clinic (baseline); T_1_, during the pre-anesthesia visit at surgical ward; T_2_, in the preoperative holding area; T_3_, at separation from parent to OR; T_4_, on entrance to OR; T_5_, the time point of ready for intravenous cannulation; VAS, Visual Analogue Scale. Values are mean (SD) or median (inter-quartile range);

^a^ *P* < 0.05 vs T_0_ within group after Bonferroni adjustment

^b^ *P* < 0.05 vs T_1_ within group after Bonferroni adjustment

^c^ *P* < 0.05 vs T_3_ within group after Bonferroni adjustment

.

The percentage of children with poor induction compliance (i.e., ICC ≥ 6) was higher in the control group compared with the picture book group [38% vs.21%; odds ratio(95%CI): 0.78(0.61–0.99); *P* = 0.041; Fig. 3B]. Parental anxiety scores at T_0_, T_1_, T_2_ and T_3_ had no significant difference between the two groups (*P* > 0.05; Table 2).

Eight percent parents thought that children feel more anxious after reading the picture book, while 70% thought it was helpful to relieve children’s anxiety. However, an overwhelming majority of parents said the picture book have no effect on themselves. Seventy-seven percent parents thought the picture book gave their children more information about the procedure (Table 3).

**Table 3:** Evaluation of the picture book by parents in the picture book group

Questions	
How many times have your child read the picture book? [*n* (0/1/2/ ≥ 3)]	5/2/12/39
Did you think the picture book comfort your child? *n* (%)	37 (70%)
Did you think the picture book stress your child out? *n* (%)	4 (8%)
Did you think the picture book comfort you? *n* (%)	11 (21%)
Did you think the picture book stress you out? *n* (%)	1 (2%)
Did you think the picture book give your child more information about the procedure? *n* (%)	41 (77%)

*n*, number of parents who answered yes to the question

## Discussion

This study demonstrated that preschool children who had an early education by reading an animated picture book illustrating surgery-related events at home prior to hospital admission exhibited significant lower anxiety level at the time ready for intravenous cannulation. Also, children in picture book group showed higher compliance rate during the anesthesia induction compared to control group. However, the anxiety of patents was not affected by reading this picture book.

Younger children undergoing anesthesia and surgery tend to show significant emotional reactions. These reactions reflect the children’s fear of being separated from their parents and familiar environment, as well as loss of control when they face the unfamiliar routines, and procedures of the hospital alone [15]. The commonly used non-pharmacological modalities include educational preparation, behavioral adaptation, parental presence at anesthesia induction, and complementary and alternative medicine [4]. An educational approach consists of providing information and preparation relevant to a child’s forthcoming surgical procedure and help families and patients to understand the process and expect favorable outcome, and it can be achieved by the tour of OR and anesthesia recovery areas, and visual depiction of perioperative events and equipment via videos or books.^4^ In our study, the children and their parents were prepared by allowing them to read an animated surgery-related picture-book at home within a week before surgery, and letting them know what would happen and develop a sense of self-control over the procedures, thereby enabling them to effectively overcome their fears and be more cooperative during anesthesia induction. Besides, information embedded in picture book was communicated via an animated character whom children have been very familiar with and would want to imitate. From our study book, the story of the lovely rabbit “Tom” would have inspired those preschoolers to face the needle bravely during intravenous injection by turning their fear into curiosity.

Children aged 3–6 years are young, immature and have short attention span, not advanced enough to recognize complicated conceptions, but they have gradually developed strong interests in stories, and understood the relationship between sentences [16]. Besides, the characteristic of cognitive development in children aged 2–7 years is to imitate and use symbols, such as symbolic play. They also gain the ability to recognize drawing or graphic images. In this age group, they learn to use language to describe their experiences and perceptions of people and things around [9]. Moreover, reported by Schultz [17], three-year-olds even have learned and developed the ability to understand the cause and effect. So we believed the picture book we used was appealing and appropriate to the development stage of young children.

A most recent and similar study demonstrated that reading an educational comic leaflet up to one week before surgery can effectively alleviate anxiety levels in children aged 6–17 years [5]. Even the picture book and the comics share the most obviously visual/ verbal features, there are major differences in the cores of definition. The picture book was specially designed for young children and it encourages young readers to visualize the scenes and take part in that social structure and environment while comics encourage readers to empathize with the specific details of the stories as they unfold and keep readers immersed in that story [16]. The character in our study book was animated as a bunny rabbit, named “Tom”, in which children are more familiar with. Engrossed young readers could image themselves participating in that scene even with some fears and worries. And moreover, the book is narrated from the perspective of children, which helps them to develop empathy and to observe and reconstruct their own concepts around children experiences. On the contrary, comic books used in other studies, is an adult’s perspective on what might happen, which could be more appropriate for older children.

There are also other educational preparation programmes described in the literature. West and colleagues [18] reported that a child life preparation session delivered on the day of surgery could significantly reduce preoperative anxiety in young children. Additionally, previous studies demonstrated that a virtual reality tour of the operating theatre one hour before surgery could effectively alleviate preoperative anxiety in children [19, 20]. Unlike these studies, the timing of intervention in our study was one week before surgery. According to our clinical experience, children’s anxiety often begins at the time of admission or even before admission, and early work reported that children who experience high levels of anxiety at one point were less likely to cooperate when undergoing procedures later [21]. From this point of view, the proactive antianxiety measures should be taken early even before hospital admission.

There are some limitations to our study. First, there is no standard scale or score system to judge how deeply the children's understanding of the picture book, so we only used verbal questions to make the best judgment as we can. Secondly, the patients were unable to be blinded out in this study, and we had ensured the assessor and anesthesiologists blinded to the group allocation. Lastly, postoperative anxiety level had not been assessed in our study, since the patients enrolled covers all types of surgery which may cause varying degree levels of postoperative anxiety.

## Conclusions

Overall, our research demonstrated that early education by reading an animated picture book depicting the story of perioperative events significantly reduced the preoperative anxiety and dramatically improved the compliance of anesthesia induction in preschool children. This approach may serve as one of the important alternatives to treat preoperative anxiety in preschoolers.

## Data Availability

The datasets used and/or analyzed during the current study are available from the corresponding author on reasonable request.

## Abbreviations

CI: Confidence interval
EASI: Emotionality, Activity, Sociability, and Impulsivity
ICC: The Induction Compliance Checklist
ITT: Intention to treat
mYPAS-SF: The modified Yale Preoperative Anxiety Scale Short Form
OR: Operating room
